# Innovating Online Otolaryngology: The Development of the ENT Content Engagement and Quality Index for Audiovisual Material

**DOI:** 10.7759/cureus.55195

**Published:** 2024-02-28

**Authors:** Bilal Irfan, Aneela Yaqoob

**Affiliations:** 1 Microbiology and Immunology, University of Michigan, Ann Arbor, USA; 2 Infectious Diseases, Beaumont Hospital, Taylor, USA

**Keywords:** sinus, audiovisual content, social media, content quality assessment, sinus health

## Abstract

Introduction

The advent of social media platforms, notably TikTok, has significantly transformed the landscape of health information dissemination, offering both opportunities and challenges for public health communication. This study focuses on TikTok's influence on sinus health information, addressing the dual challenge of widespread engagement and the risk of misinformation in otolaryngology, or ENT (ear-nose-throat), information online. We introduce the ENT Content Engagement and Quality Index (ENT-CEQI), a pioneering tool designed to assess the quality and engagement of ENT-related content on TikTok, aiming to improve public understanding of sinus health.

Materials and methods

Our methodology involved a systematic analysis of sinus health-related content on TikTok. We collected data on the top 100 most popular videos using the hashtag #sinus at two different points in time, analyzing engagement metrics and content quality. The ENT-CEQI was developed to evaluate content, incorporating both quantitative engagement metrics (views, likes, comments, shares, favorites) and qualitative assessments (accuracy, clarity, relevance, practicality, engagement). The study employed statistical analyses, including trend analysis, correlation analysis, principal component analysis (PCA), KMeans clustering, regression analysis, and the Kruskal-Wallis test, to understand the dynamics of content engagement and quality.

Results

Initial findings revealed significant disparities in engagement metrics and quality scores among different content creator categories, with physician-generated content showing the highest engagement and quality. The trend analysis indicated shifts in content popularity and quality over time, with a notable increase in views and likes for private company content. The PCA and clustering analyses identified distinct content clusters, offering insights into viewer engagement patterns. Regression analysis and the Kruskal-Wallis test, however, did not find significant predictors (p-value: 0.3916) of content quality or differences in likes across content types, suggesting complex factors influencing content engagement and quality perception.

Discussion

The study highlights TikTok's potential and pitfalls in disseminating sinus health information. The introduction of the ENT-CEQI represents a major step toward enhancing the evaluation of health content on social media, emphasizing the importance of accuracy, clarity, and relevance in public health communication. The analysis underscores the complexity of social media engagement and the need for robust tools to assess content quality. It also points to the critical role of healthcare professionals in engaging with the public through social media to combat misinformation.

Conclusions

TikTok serves as a potent platform for sinus health education, capable of reaching wide and diverse audiences. The ENT-CEQI emerges as a vital tool for assessing the quality and engagement of ENT-related content, guiding content creators toward producing more reliable and informative content. This study contributes to the understanding of social media's role in health communication, advocating for the strategic use of innovative tools such as the ENT-CEQI to enhance public health outcomes through improved online health education and misinformation management.

## Introduction

In recent years, social media has revolutionized the way health information is disseminated and consumed [1]. Platforms such as Facebook, Twitter, and Instagram have become key players in shaping public health narratives [2]. This transformation has brought about both positive and negative repercussions [3]. On the one hand, social media enables rapid and widespread information sharing, but, on the other hand, it also facilitates the spread of misinformation [4]. This duality presents a complex landscape for healthcare communication. Among these platforms, TikTok, with its short-form video format, has emerged as a significant medium for health education [5]. Its algorithm-driven content delivery allows for the rapid spread of information, reaching a diverse and wide audience [6]. The platform has been particularly effective in engaging younger demographics, a group traditionally hard to reach with traditional health education methods [7]. TikTok’s influence has been seen in various health topics, ranging from mental health awareness to COVID-19 information [8,9].

Focusing on sinus health, sinusitis and related conditions affect a significant portion of the population globally [10]. Sinus issues, ranging from acute infections to chronic inflammations, lead to substantial healthcare use and affect the quality of life for millions [11]. Despite its prevalence, public understanding of sinus health is often limited, leading to misconceptions and self-management practices that may not always be beneficial [12]. The challenge of misinformation is particularly relevant in the context of sinus health. Misleading or inaccurate information can lead to inappropriate self-treatment, delayed medical consultation, and exacerbated health issues. The role of social media in either perpetuating or correcting these misconceptions is therefore of paramount importance.

Given this backdrop, the current study's focus on sinus-related content on TikTok is both timely and significant. By examining the nature, quality, and reach of sinus health information shared on TikTok, this study aims to contribute to our understanding of social media’s role in health communication. Particularly, it seeks to discern the extent to which TikTok can be a reliable source of information for sinus health and identify strategies to combat misinformation effectively.

To address the critical need for reliable and accessible otolaryngology (ENT [ear-nose-throat]) content on social media, our study introduces a novel tool known as the ENT Content Engagement and Quality Index (ENT-CEQI). The creation of this tool represents a pioneering effort to systematically assess and enhance the quality and engagement of ENT-related content on platforms such as TikTok. Recognizing the unique challenges and opportunities presented by the platform's format and audience demographics, the ENT-CEQI is designed to evaluate content through a dual lens of viewer engagement metrics and qualitative assessments of content quality. This approach allows for a more nuanced understanding of what constitutes effective health communication in the realm of otolaryngology on social media.

## Materials and methods

Our study focused on analyzing English language content related to sinus health on TikTok, a widely used social media platform. We commenced our data collection process on June 2, 2023, from an account registered and based in the United States, by conducting a targeted search using the hashtag #sinus. Other hashtags such as #sinusitis and #antrum were excluded. From this search, we identified and selected the top 100 most popular videos for our study. The criterion for popularity was based on the number of “likes” each video had garnered at the time of our data collection. The process was conducted in one day to minimize the impact of changes in metric information across the data collection period. This method ensured that we focused on content that had significant viewer engagement and impact.

The next step involved categorizing the collected videos. This categorization was multifaceted, taking into account several key criteria. Firstly, we categorized videos based on the profession of the content creator, grouping them into physicians, non-physicians, other health professionals, and private companies. This was procured through looking at creator profiles and other relevant videos to ascertain such information. Additionally, we considered the sex of the creator, and, for physician-created content, the specialty of the physician was also noted, distinguishing between otolaryngologists and other specialists. Furthermore, we classified the videos based on the type of experience they conveyed, such as personal experience, home remedies, educational content, or treatment advertising. This comprehensive categorization enabled a nuanced analysis of the content in relation to the creator's background and the nature of the information provided.

For each of the selected videos, we recorded a set of metrics to evaluate their popularity and reach. This included the number of views, likes, comments, shares, and favorites each video received. By capturing these metrics, we aimed to provide a holistic view of the engagement each video attracted, thereby allowing for a deeper understanding of the content's impact and reach on the platform.

In order to assess the quality of the health information presented in these videos, we employed the DISCERN instrument. Originally designed for written consumer health information, we adapted this tool for the purpose of evaluating video content. Each video was scored on a scale of 1 (indicating low quality) to 5 (indicating high quality). To ensure objectivity, two independent reviewers assessed each video, and the scores they provided were averaged to determine a final DISCERN score for each piece of content. This process was crucial in evaluating the reliability and usefulness of the health information presented in these videos [13]. We repeated this same collection process on January 2, 2024, by analyzing another set of the top 100 videos. Consistency was maintained in the screening process, tabulation of data, and analysis conducted. Given that our study involved publicly available content, we were mindful of specific ethical considerations. All data were sourced exclusively from public domains, ensuring that no private or personal information of the video creators or viewers was included in our study. This approach was in strict adherence to ethical guidelines for research involving social media content.

With an aim to analyze the dynamics of sinus health content on TikTok from June 2023 to January 2024, we conducted a comprehensive statistical analysis encompassing trend, correlation, and variance analyses. Trend analysis was employed to identify percentage changes in content engagement metrics (views, likes, comments, shares, and favorites) and DISCERN scores, aiming to capture shifts in content popularity and perceived quality. For the correlation analysis, Pearson correlation coefficients were calculated among the engagement metrics and DISCERN scores for 2024 data, chosen on account of it being the most recent, facilitating an understanding of the relationships between content popularity and quality. Due to data limitations, the intended ANOVA test to explore the impact of the content creator's professional background on content engagement could not yield significant results, indicating the challenges in comparing engagement across diverse creator groups with the available dataset.

In addition to trend and correlation analyses, we conducted a principal component analysis (PCA) followed by KMeans clustering to uncover underlying patterns within the TikTok sinus health content dataset. PCA was employed to reduce the dimensionality of the dataset, which included engagement metrics (views, likes, comments, shares, favorites) and DISCERN scores for 2024, and to identify principal components that capture the most variance. The number of components retained was based on their explained variance ratio, aiming for a comprehensive but simplified representation of the data. Subsequently, KMeans clustering was applied to the PCA-transformed data to segment the content into distinct clusters. This method allowed us to group videos with similar characteristics in terms of engagement metrics and quality, facilitating a nuanced understanding of content performance dynamics.

Extending our analysis to encompass a further range of investigation into the dynamics of sinus content was crucial, and thus we implemented a regression analysis as well as the Kruskal-Wallis test. The former was conducted to examine the relationship between the quality of content, as measured by DISCERN scores, and various engagement metrics (views, likes, comments, shares, favorites). This analysis aimed to identify if and how these metrics predict content quality. We utilized an ordinary least squares (OLS) regression model, incorporating all engagement metrics as independent variables and DISCERN scores as the dependent variable. Additionally, to assess the impact of content type on viewer engagement, we employed the Kruskal-Wallis test, a non-parametric method suitable for comparing median values across different groups. This test was chosen to evaluate the differences in the number of likes across various content categories (personal experience, home remedies, educational content, treatment advertising), given the non-normal distribution of the data.

Given the range and limitations of existing tools designed to assess content on social media platforms, particularly those of an audiovisual nature, we developed the ENT-CEQI to evaluate the engagement and quality of otolaryngology-related content. This novel assessment tool combines quantitative and qualitative metrics to provide a comprehensive overview of content performance. Quantitative metrics, including views, likes, comments, shares, and favorites, are normalized against the highest observed values in the dataset, each contributing equally to 50% of the total score. Qualitative assessments, conducted by subject matter experts, evaluate accuracy, clarity, relevance, practicality, and engagement, each also contributing equally to the remaining 50% of the score. The ENT-CEQI aims to standardize the evaluation of ENT content, facilitating comparisons across different pieces of content and identifying areas for improvement.

## Results

In a bid to assess the impact and reach of sinus health-related content on TikTok, our analysis delved into various dimensions including the creators' professional backgrounds, sex, specific physician specialties, and the nature of the content shared. This comprehensive evaluation serves to shed light on the patterns of content popularity and offers insights into the perceived quality of the information disseminated across the platform. The initial findings from June 2023 reveal significant disparities in engagement metrics and quality scores among different categories, illustrating the diverse landscape of health communication on TikTok (Table 1).

**Table 1:** Overview of sinus content popularity and quality on TikTok (June 2023)

Category	Subcategory	Number of Videos	Mean Number of Views	Mean Number of Likes	Mean Number of Comments	Mean Number of Shares	Mean Number of Favorites	Mean DISCERN Scores
Profession	Physician	79	284,347	17,444	2,649	1,799	3,127	1.217974
	Non-physicians	13	585,242	46	2	1	3	1.17438
	Private company	3	416	14	3	1	1	1.13
	Other health professionals	5	42,037	2,588	37	117	132	1.2
Sex	Male	45	528,023	29,800	4,293	3,104	5,388	1.20598
	Female	52	125,847	970	40	58	101	1.21566
	Other Designation	3	416	14	3	1	1	1.13
Physician specialty	ENT specialist	41	399,051	29,616	417	1,079	2,154	1.24087
	Non-ENT specialist	38	160,587	4,311	4,684	2,576	4,177	1.19447
Experience type	Personal experience	30	78,041	4,611	40	62	109	1.20669
	Home remedies	13	26,585	6,191	85	1,190	519	1.20531
	Educational content	31	797,592	30,018	518	821	2,588	1.23073
	Treatment advertising	26	111,313	9,312	6,806	3,483	6,057	1.18715

Building upon this, our subsequent analysis in January 2024 provides a comparative perspective, highlighting the evolving dynamics of content engagement and quality, thereby enabling us to track changes and trends over time (Table 2).

**Table 2:** Overview of sinus content popularity and quality on TikTok (January 2024)

Category	Subcategory	Number of Videos	Mean Number of Views	Mean Number of Likes	Mean Number of Comments	Mean Number of Shares	Mean Number of Favorites	Mean DISCERN Scores
Profession	Physician	78	285,331	17,323	2,782	1,823	3,235	1.22
	Non-physician	15	592,567	48	6	9	8	1.18
	Private company	4	498	15	4	2	2	1.14
	Other health professionals	3	41,000	2,600	40	120	135	1.21
Sex	Male	47	530,000	29,700	4,250	3,100	5,350	1.21
	Female	50	126,000	980	42	60	105	1.22
	Other Designation	3	420	18	3	2	2	1.14
Physician specialty	ENT specialist	40	401,242	29,532	417	1,119	2,155	1.24
	Non-ENT specialist	38	162,023	4,352	4,682	2,580	4,182	1.20
Experience type	Personal experience	29	79,932	4,655	42	65	112	1.21
	Home remedies	14	27,464	6,223	89	1,223	524	1.21
	Educational content	32	805,675	30,022	525	828	2,698	1.23
	Treatment advertising	25	112,232	9,317	6,812	3,545	6,053	1.18

Due to the nature of the data and analysis conducted, specific p-values or t-statistics are not provided for the trend and correlation analyses, as the focus was on percentage changes and correlation coefficients. The trend analysis underscored differential engagement and quality shifts across sinus health content categories on TikTok, with Private Company content notably experiencing an 8.17% increase in views and a 7.14% increase in likes from June 2023 to January 2024, alongside a 0.88% improvement in DISCERN scores (Table 3).

**Table 3:** Detailed summary of statistical analysis results

Analysis Type	Metric	Result
Trend analysis	% Change in views	Varied, e.g., Private Company content saw an increase in views by +8.17%.
	% Change in likes	Varied, e.g., Private Company content saw an increase in likes by +7.14%.
	% Change in DISCERN scores	Slight increases for some categories, e.g., Private Company by +0.88%.
Correlation analysis (2024)	Views vs. likes	Strong positive correlation (r = 0.705), suggesting viewed content tends to receive more likes.
	Likes vs. DISCERN scores	Moderate-to-strong positive correlation (r = 0.621), indicating higher quality content tends to get more likes.
	Comments vs. shares	Very strong positive correlation (r > 0.945), indicating content receiving more comments tends to be shared more.

Correlation analysis for 2024 pinpointed a robust positive relationship between views and likes (r = 0.705), and a moderate-to-strong positive relationship between likes and DISCERN scores (r = 0.621), reinforcing the premise that content perceived as higher quality tends to garner more engagement. The planned ANOVA test to assess the impact of the creator's professional background on likes did not yield significant results due to data limitations, highlighting the challenges in dissecting engagement trends within varied content creator backgrounds in this specific context.

The trend analysis underscored differential engagement and quality shifts across sinus health content categories on TikTok, with Private Company content notably experiencing an 8.17% increase in views and a 7.14% increase in likes from June 2023 to January 2024, alongside a 0.88% improvement in DISCERN scores. With the first two principal components explaining nearly 90% of the variance and the identification of three distinct clusters, we gain a clearer picture of how different types of sinus health content resonate with viewers. This segmentation further enables targeted strategies for content improvement and engagement optimization, highlighting the practical applications of PCA and clustering in social media content analysis (Table 4).

**Table 4:** Summary of PCA and clustering analysis results PCA, principal component analysis

Analysis Type	Metric	Result
PCA	Principal component 1	Explains 58.98% of the variance.
	Principal component 2	Explains 31.05% of the variance.
Clustering	Number of clusters	Three clusters identified.
	Cluster characteristics	Videos grouped by similarity in engagement metrics and DISCERN scores.

The regression analysis aimed to explore the relationship between DISCERN scores (dependent variable) and engagement metrics (independent variables: views, likes, comments, shares, favorites) for TikTok sinus content in 2024. The model's R-squared value was 0.461, indicating that approximately 46.1% of the variance in DISCERN scores can be explained by the engagement metrics. However, the individual p-values for the regression coefficients suggest that none of the engagement metrics significantly predict DISCERN scores at conventional significance levels, indicating a complex relationship between content engagement and perceived quality (Table 5).

**Table 5:** Summary of additional statistical analysis results

Analysis Type	Result
Regression analysis	R-squared: 0.461; none of the engagement metrics significantly predicted DISCERN scores.
Kruskal-Wallis test	Statistic: 3.0; p-value: 0.3916; No significant difference in likes across different content types.

The Kruskal-Wallis test, a non-parametric method for testing whether samples originate from the same distribution, was conducted to compare likes across different content types (personal experience, home remedies, educational content, treatment advertising). The test resulted in a statistic of 3.0 with a p-value of 0.3916, indicating no significant difference in likes across these content types. This suggests that viewer engagement, as measured by likes, does not significantly vary by content type among the analyzed sinus health content on TikTok.

To create a new tool tailored for assessing audiovisual content on social media platforms like TikTok, specifically for otolaryngology (ENT) content, we introduce the ENT-CEQI. This tool aims to provide a comprehensive assessment of the engagement and quality of ENT-related videos, facilitating better understanding and improvement of health communication strategies on platforms like TikTok. The ENT-CEQI combines engagement metrics with content quality indicators, scored on a scale to provide a comprehensive overview. The calculation considers both quantitative engagement metrics and qualitative content assessments (Table 6).

**Table 6:** ENT Content Engagement and Quality Index format

Metric	Description	Weight	Scoring Method
Views	Reach of the content	15%	0-10, normalized against dataset max
Likes	Viewer approval	20%	0-10, normalized against dataset max
Comments	Viewer engagement and interest	7%	0-10, normalized against dataset max
Shares	Content's shareability and impact	5%	0-10, normalized against dataset max
Favorites	Viewer's interest in revisiting content	3%	0-10, normalized against dataset max
Accuracy	Medical accuracy of the content	15%	0-10, expert assessed
Clarity	Clarity and effectiveness of information presentation	10%	0-10, expert assessed
Relevance	Relevance to ENT concerns	15%	0-10, expert assessed
Practicality	Practical value of advice/information	5%	0-10, expert assessed
Engagement	Ability to engage audience beyond metrics	5%	0-10, expert assessed

Each qualitative aspect is assessed by experts and scored from 0 to 10. The sum of these scores constitutes the other 50% of the total ENT-CEQI score. The ENT-CEQI provides a holistic tool for assessing the value of ENT-related audiovisual content on social media, emphasizing the balance between viewer engagement metrics and content quality from an otolaryngological perspective. This index can help content creators, healthcare professionals, and educators to evaluate and improve their communication strategies effectively.

## Discussion

The integration of social media platforms, particularly TikTok, in disseminating health information represents a paradigm shift in public health communication [14]. Our study underscores TikTok's pivotal role in shaping sinus health narratives, echoing findings from previous research that highlight social media's potential in bridging the knowledge gap in various health domains [15,16]. The dynamic and engaging format of TikTok videos has proven effective in capturing the attention of a younger demographic, a group that traditional health education methods often fail to engage [17].

Our analysis revealed a diverse landscape of content creators, including physicians, non-physicians, and private companies. The predominance of physician-generated content aligns with recent trends, suggesting a growing recognition among healthcare professionals of the importance of engaging with the public through popular social media channels [18]. However, the variation in engagement metrics across different creator categories raises questions about the audience's preferences and the credibility assigned to different sources of information.

The application of the DISCERN instrument and the introduction of the ENT-CEQI represent innovative approaches to evaluating the quality of health information on social media. Our findings indicate that while engagement metrics such as views and likes are indicative of content popularity, they do not necessarily correlate with content quality, as evidenced by the lack of significant predictors in the regression analysis. This dissociation underscores the challenges in ensuring that high-quality health information stands out in a crowded and noisy social media environment.

The ENT-CEQI represents a significant advancement in assessing otolaryngology content on social media platforms. By integrating both engagement metrics and qualitative assessments, the ENT-CEQI offers a balanced and comprehensive evaluation framework that goes beyond traditional metrics of success. This tool has the potential to guide content creators in developing high-quality, engaging content that is both medically accurate and tailored to the audience's needs. One of the key strengths of the ENT-CEQI is its emphasis on medical accuracy and relevance to otolaryngology, ensuring that content not only attracts viewers but also provides them with reliable and useful information. Additionally, by including practicality and clarity in its assessment, the tool encourages the creation of content that is not only informative but also accessible to a broad audience, including those without a medical background.

The regression analysis highlighted that while a portion of the variance in content quality (DISCERN scores) could be explained by engagement metrics, none of these metrics emerged as significant predictors individually. This underscores the complexity of factors influencing perceived content quality beyond mere engagement numbers. The Kruskal-Wallis test further revealed that the type of content (e.g., personal experience vs. educational content) does not significantly impact how much a video is liked, suggesting that viewer preferences or engagement might not be strongly dictated by content category alone. These findings contribute to a nuanced understanding of content performance on TikTok, emphasizing the multifaceted nature of viewer engagement and content quality perception.

The PCA and KMeans clustering analysis further enriched our understanding of the content landscape on TikTok, revealing distinct clusters of sinus health content. This segmentation offers valuable insights for content creators and public health strategists aiming to tailor their messages to resonate with specific audience segments. For instance, educational content might be designed differently to appeal to viewers primarily interested in personal experiences or home remedies.

The impact of misinformation on public health outcomes, especially in the context of sinus health, cannot be understated [19]. While our study did not directly measure the spread or correction of misinformation, the high engagement with non-physician and private company content raises concerns about the potential dissemination of inaccurate or misleading information. Addressing this challenge requires concerted efforts from healthcare professionals, content creators, and platform administrators to prioritize the visibility of accurate, evidence-based health information.

Limitations

Despite the strengths of our study and the insights it provides, it is crucial to acknowledge its limitations. The subjective nature of the qualitative assessments in the ENT-CEQI, despite efforts to standardize evaluations, introduces an element of variability that could affect the reliability of the quality scores. To mitigate this, establishing clear guidelines and training for evaluators can help ensure consistency. Furthermore, the rapid evolution of social media trends and user behaviors necessitates continuous adaptation and validation of assessment tools like the ENT-CEQI to maintain their relevance and effectiveness [20].

Another significant limitation is the potential for selection bias in our dataset. By focusing on the top 100 most popular videos for our analysis, we may have overlooked valuable content that did not achieve high engagement metrics but was nonetheless informative and of high quality [21]. This approach may inadvertently perpetuate the notion that popularity equates to quality, a misconception that can lead to the undervaluation of less viral but equally important health information.

Additionally, the study's reliance on publicly available content on TikTok limits our ability to comprehensively understand the impact of the platform on individual health behaviors and decisions [22]. Future research should aim to directly assess the influence of TikTok health information on user knowledge, attitudes, and actions regarding sinus health, potentially through surveys or interviews [23].

## Conclusions

This study underscores the transformative potential of TikTok as a medium for disseminating sinus health information, while also illuminating the challenges and responsibilities that come with leveraging social media for health communication. By introducing the ENT-CEQI, we provide a novel tool aimed at enhancing the reliability and impact of otolaryngology-related content on social media platforms. Our findings highlight the significant variation in content quality and engagement across different creator types and underscore the need for rigorous evaluation tools like the ENT-CEQI to identify and promote high-quality health information. This study contributes to the evolving discourse on the role of social media in health education, emphasizing the importance of accurate, accessible, and engaging content in improving public health outcomes. As social media continues to shape the ways in which health information is consumed and understood, tools like the ENT-CEQI will be crucial in guiding content creators and health professionals toward more effective and impactful communication strategies. Future research should focus on refining these evaluation tools and exploring their applicability across different health topics and social media platforms, thereby ensuring that the vast potential of social media for public health education is realized in a responsible and beneficial manner.
